# Association of Salivary Micro Ribonucleic Acid Levels With the Severity of Severe Acute Respiratory Syndrome Coronavirus 2 Infection in Children

**DOI:** 10.1002/pdi3.70050

**Published:** 2026-06-17

**Authors:** Steven D. Hicks, Dongxiao Zhu, Nirupama Kannikeswaran, Kathleen Meert, Wei Chen, Rhea Sullivan, Srinivasan Suresh, Usha Sethuraman

**Affiliations:** ^1^ Department of Pediatrics Pennsylvania State University Medical Center Harrisburg Pennsylvania USA; ^2^ Department of Computer Science Wayne State University Detroit Michigan USA; ^3^ Division of Emergency Medicine Department of Pediatrics Children's Hospital of Michigan Central Michigan University Detroit Michigan USA; ^4^ Division of Critical Care Department of Pediatrics Children's Hospital of Michigan Central Michigan University Detroit Michigan USA; ^5^ Population Science Department of Oncology Wayne State University School of Medicine Detroit Michigan USA; ^6^ Department of Pediatrics UPMC Children's Hospital of Pittsburgh University of Pittsburgh Pittsburgh Pennsylvania USA

## Abstract

Severe acute respiratory syndrome coronavirus 2 (SARS‐CoV‐2) may impact immune‐related micro ribonucleic acids (miRNAs). The role of salivary miRNAs as predictors of severe infection in children is unknown. This study sought to examine the relationship between SARS‐CoV‐2 severity and salivary miRNA levels in children. A convenience sample of 400 children with SARS‐CoV‐2 infection was prospectively evaluated between March 2021 and February 2022 at two U.S. children's hospitals. Saliva swabs were obtained. Levels of 3 miRNAs previously implicated in SARS‐CoV‐2 pathophysiology were measured with quantitative polymerase chain reaction (miR‐1273c, miR‐296‐5p, and miR‐4495). The primary outcome measure was the occurrence of severe illness, including respiratory failure, shock, or death from a related cause within 1 month of evaluation. Disease resolution was determined through chart review and caretaker phone calls 4 weeks after the initial visit. Saliva samples from 395 (severe = 105) participants (mean age: 7.4 ± 5.9 years, 51.7% female, 54.7% African American) were analyzed. Children with severe outcomes had lower levels of miR‐296‐5p (fold difference: 0.361, *d* = 0.111, *p* = 0.046). Multivariable adjusted logistic regression analysis demonstrated an inverse association between severe outcomes and levels of miR‐296‐5p (adjusted odds ratio = 0.89; 95% confidence interval [0.81, 0.98], *p* = 0.021) while controlling for age, sex, race, weight, insurance, diabetes, asthma, fever, and symptom duration. The model had an area under curve of 0.744 (sensitivity = 0.71, specificity = 0.66). Measurement of saliva miRNA in conjunction with demographic and clinical characteristics may aid in the prediction of severe illness. Larger, longitudinal studies to assess the utility of salivary miRNAs in SARS‐CoV‐2 and other viral infections are needed.

## Graphical abstract

This cohort study of 395 children (mean age 7 years) with SARS‐CoV‐2 demonstrates that measuring levels of miR‐296‐5p in saliva at the time of initial clinical presentation may aid the prognosis of severe symptoms.

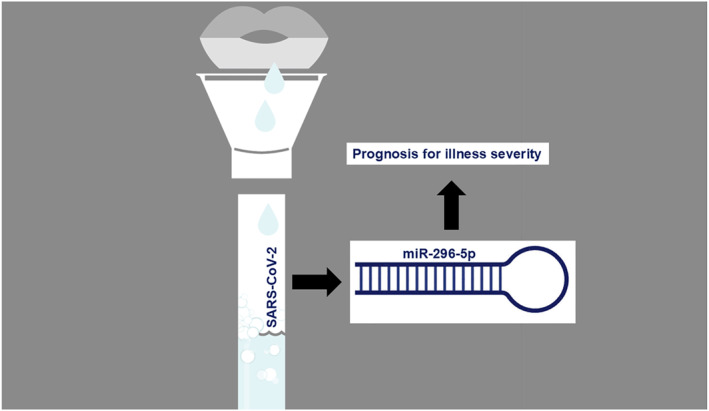

## 1 Introduction

The coronavirus disease 2019 (COVID‐19) pandemic due to severe acute respiratory syndrome coronavirus 2 (SARS‐CoV‐2) has caused over 1.1 million deaths in the Detroit, Michigan and Pittsburgh, Pennsylvania, United States [1]. Nearly 15.6 million children tested positive for the virus, with illness severity ranging from mild to severe [2, 3, 4, 5, 6]. Examples of severe illness in children included respiratory distress, endocrine dysfunction, and multisystem inflammatory syndrome in children (MIS‐C). Although lower than in adults, one in four hospitalized children required critical care in 2022, with 2.3% needing mechanical ventilation [7, 8]. The ability of front‐line physicians to recognize severe illness and determine appropriate disposition is important for optimal health outcomes. However, this determination was challenging because early SARS‐CoV‐2 symptoms could be subtle and mirror other viral infections. The development of objective biomarkers to define SARS‐CoV‐2 severity could aid clinical decision‐making, but currently, no biologic test to predict illness severity in children exists [4, 5, 6, 9, 10].

Interactions between the host immune system and pathogens can lead to epigenetic modifications that allow a pathogen to evade immune detection and cause severe symptoms [11, 12, 13]. Epigenetic modifications may alter immune function by impeding transcription or translation of key signaling cascades [14, 15, 16]. A well‐known epi‐transcriptional process involves transcriptional repression by micro ribonucleic acids (miRNAs). Perturbations in miRNA activity may contribute to a variety of disease processes and are touted as emerging biomarkers for diagnosis, prognosis, and therapy [17, 18, 19, 20, 21, 22, 23, 24, 25, 26, 27, 28, 29, 30, 31]. A study of adults with SARS‐CoV‐2 infection found that human miRNAs may bind to key elements within the SARS‐CoV‐2 genome [32]. Through this host interaction, SARS‐CoV‐2 may evade immune detection by sponging critical immune‐related miRNAs [33]. In this manner, the virus may dysregulate host immune responses, enhancing viral replication and causing severe illness. This finding led to numerous studies examining the utility of miRNAs as biomarkers for COVID‐19 [34, 35, 36, 37, 38, 39, 40, 41, 42]. Most studies involved miRNAs in the blood of adult patients. However, saliva contains robust concentrations of immune‐related miRNAs and serves as the primary interface between the host and SARS‐CoV‐2 [43]. In addition, the ability of clinical personnel to rapidly collect saliva in outpatient and urgent care settings makes it an attractive biofluid for biomarker discovery [44].

Recently, we showed that the levels of 43 salivary miRNAs (of the 1606 miRNAs measured in saliva) were altered in children with severe outcomes due to SARS‐CoV‐2 infection, and the majority were downregulated [45]. Candidate miRNAs displaying the most robust associations with SARS‐CoV‐2 severity included miR‐296‐5p, miR‐1273c, and miR‐4495. The prior study, however, involved a small sample size, used expensive, time‐consuming RNA sequencing, and lacked confirmatory testing with quantitative polymerase chain reaction (qPCR).

The goals of the present study were to (1) confirm the relationship between previously identified saliva miRNA candidates and SARS‐CoV‐2 severity in children using a clinically adaptable platform (i.e., qPCR) and (2) examine the clinical utility of salivary miRNAs for predicting the severity of SARS‐CoV‐2 illness in pediatric patients. We hypothesized that a subset of immune‐related miRNAs would display lower salivary levels in pediatric patients with severe SARS‐CoV‐2 symptoms. The results of this research could help identify novel therapeutic targets for SARS‐CoV‐2 and eventually lead to a molecular test predicting SARS‐CoV‐2 severity.

## 2 Methods

This prospective study utilized a convenience sample of children (≤ 18 years of age) prospectively enrolled at the time of evaluation in the emergency department (ED) of two children's hospitals between March 2021 and February 2022. A standardized protocol for SARS‐CoV‐2 testing was employed at both EDs, involving rapid qPCR with additional serology testing when clinically indicated. This study was approved by the University of Pittsburgh Medical Center (UPMC, Detroit Children's Hospital and Pittsburgh Medical Center) Institutional Review Board (IRB), which acts as a central IRB (STUDY21010046). Parents or legal guardians gave written, informed consent, and children provided written assent when applicable.

### 2.1 Study Definitions

1. Infection with SARS‐CoV‐2 was defined by a positive reverse transcriptase polymerase chain reaction (RT–PCR) test, positive serology (SARS‐CoV‐2 IgG), or an epidemiological link.
2. Severe SARS‐CoV‐2 symptoms were defined using modified criteria from the Centers for Disease Control and Prevention [46] as the presence of one or more of the following conditions: respiratory failure (requiring supplemental oxygen [≥ 50% FiO_2_], or noninvasive or invasive positive pressure ventilation), shock (requiring extracorporeal membrane oxygenation, vasopressors, or inotropes), or death from a related cause during hospitalization or within 1 month of discharge. Severity status was determined by two clinicians, blinded to molecular results, by examining medical records and parent/guardian surveys from 30 days postdischarge. Any disagreements in severity status were resolved by a third investigator.
3. Length of stay (LOS) was defined as the time (in days) from hospital ED arrival to hospital discharge.
4. The hospitalization rate was defined as the proportion of children in the cohort of participants who were admitted to the hospital.

### 2.2 Inclusion Criteria

Children ≤ 18 years of age who presented during study hours for clinical evaluation, were accompanied by a legal caretaker, and were diagnosed with SARS‐CoV‐2 infection.

### 2.3 Exclusion Criteria

Pregnancy, trauma to the mouth, head, or neck, active seizures, dental infection, presentation for psychiatric evaluation, or being accompanied by a non‐English‐ or non‐Spanish‐speaking parent or guardian led to the exclusion.

### 2.4 Saliva Samples and Study Data

The research team was trained to employ standardized procedures for saliva sample collection using video instructions provided by the kit manufacturer (Genotek, Kanata, ON, Canada). After a tap water rinse, saliva samples were collected by placing a highly absorbent swab in the sublingual and parotid regions for 10–20 s and then submerging the swab in nucleic acid stabilization reagent (Catalog #: ORE‐100, Genotek, Kanata, ON, Canada). The sample was mixed with the stabilizer in the tube by gentle shaking and stored at room temperature. The samples were shipped on the first business day of each month to the genomics core facility for processing.

Clinical data were concatenated by two trained research assistants into a REDCap [47, 48] data collection platform (v 11.4.4). All data were verified by a third research assistant. Any data errors were rechecked and validated by the data extractor. The parents or guardians of all participants were contacted by phone 7 and 30 days after discharge to complete surveys about their child's symptoms (symptom resolution, worsening, evolution, or return visits and related outcomes). Up to five phone calls were completed in an attempt to reach each participant's parent or guardian. If the study team was unable to reach the parent or guardian after five attempts on consecutive days, no further attempts were made, and severity was assessed using only medical records. As such, complete demographic data were not available for two participants. However, because these two participants had verified primary outcome assessments (i.e., SARS‐CoV‐2 severity) and salivary RNA data, they were retained for the final analysis.

### 2.5 Biomarker Assessments: Saliva Processing

RNA extraction was completed with the miRNeasy Kit (QIAGEN, Germantown, MD, United States). As previously reported [45], high‐throughput nucleic acid sequencing was used to measure 1606 human miRNAs in the saliva of 197 children with confirmed SARS‐CoV‐2. Of the 43 miRNAs that displayed a difference in the saliva of children with severe symptoms (fold change > 2.0, adjusted *p* < 0.05), three candidates (miR‐1273c, miR‐296‐5p, and miR‐4495) were selected for validation in this study. Criteria for candidate selection included (1) physiologic relevance to the human immune response (based on gene ontology analysis) and (2) robust expression across all saliva samples (based on raw read count data). For this study, amplification of the 3 miRNA candidates was achieved with custom primers and a standard protocol involving the MistiCq system (Sigma Aldrich, Allentown, PA, United States). Relative miRNA quantification was completed through qPCR on a CLIA‐approved QuantStudio 5 machine (Applied Biosystems, Waltham, MA, United States) at the Penn State Genome Sciences Core Facility. All qPCR reactions involved 300 nm of a custom primer and 2 ng of cDNA template, with SYBR green wavelength detection in a 3‐step, 40‐cycle procedure (denaturation at 95°C for 3 min, annealing for 20 s, extension at 72°C for 60 s). No‐template controls were used for each primer, melt curve analyses were run for all reactions, and each sample was measured in triplicate. Samples that failed to meet quality control (QC) standards (mean cycle threshold [Ct] value > negative control; Ct standard deviation > 1.0; melt curve consistent with primer dimer) were reanalyzed. Samples that failed QC analysis on a second run were excluded from downstream statistical analysis (*n* = 5). Levels of the three candidate miRNAs were expressed using the delta‐Ct method relative to miR‐29c‐5p. This “control” miRNA was chosen for its consistent presence in human saliva (present in 99% of samples), stable expression across participants (coefficient of variation: 105), and lack of variation between children with severe and nonsevere SARS‐CoV‐2 symptoms (*p* = 0.70) in a prior study [45]. Sequences for the custom primers (Integrated DNA Technologies, Coralville, IA, United States) were as follows: miR‐29c‐5p (FWD: TGACCGATTTCTCCTGG, REV: CCAGTTTTTTTTTTTTTTTGTCATG), miR‐1273c (FWD: GGCGACAAAACGAGACC, REV: GTCCAGTTTTTTTTTTTTTTTCTCACG), miR‐4495 (FWD: CGCAGAATGTAAACAGGCTTTT, REV: CAGGTCCAGTTTTTTTTTTTTTTTACGA), and miR‐296‐5p (FWD: GGGCCCCCCCTCAAT, REV: CAGGTCCAGTTTTTTTTTTTTTTACTGG).

### 2.6 Statistics

The study's primary outcome was severe symptoms from SARS‐CoV‐2 infection. The secondary outcome was hospital LOS (in days). Phenotypic features were compared between children with severe and nonsevere symptoms using chi‐squared tests for dichotomous features, analysis of variance (ANOVA) for other categorical features, and Student's *t* tests for continuous features. The miRNAs measured with qPCR were evaluated in the saliva samples of 395 of the 400 children (5 samples were excluded due to inadequate volumes or poor quality). Logistic regression was used to assess the association between miRNAs and disease severity, and Poisson regression was used to assess zero‐inflated LOS. Multivariable regression models included covariates such as age, sex, race, weight, insurance status, presence of diabetes, asthma, or fever, and symptom duration at the time of saliva collection, based on a priori knowledge of severity risks [49]. Stepwise selection for a regression model with Bayesian information criteria was performed for SARS‐CoV‐2 severity, the study's primary objective. Sample size determinations were based on SARS‐CoV‐2 infection rates at the participating institutions during the study period and the anticipated feasibility of accruing participants. We estimated that 400 participants would be enrolled from both sites and that 80 (20%) of them would have severe outcomes. Based on these estimations, a logistic regression with 80 samples from patients with severe symptoms would be able to accommodate a model with 8 predictors (10 samples per predictor). R version 4.1.0 was used for statistical analyses [50].

## 3 Results

### 3.1 Participants

**FIGURE 1:**
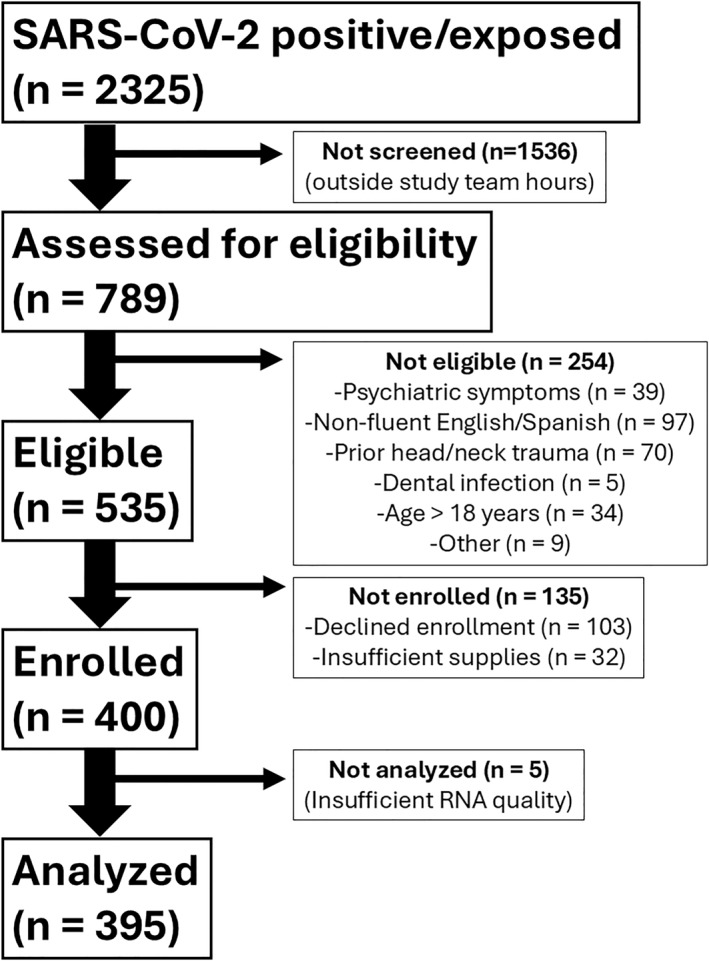
Enrollment diagram for participants in the SPITS‐MISC study. The flow diagram displays eligible, screened, and enrolled participants, culminating in 395 participants with saliva miRNAs analyzed for this study. SARS‐CoV‐2, severe acute respiratory syndrome coronavirus 2; SPITS‐MISC, Severity Predictors Integrating Salivary Transcriptomics and proteomics with Multi neural network Intelligence in SARS‐CoV2 infection in Children.

There were 400 children enrolled. Saliva samples from 395 participants (severe = 105) met quality criteria for analysis (Figure 1). The average participant age was 7.4 ± 5.9 years, 203 (51.4%) were female, and 215 (54.4%) were African American. Of the total participants, 292 (73.9%) were covered by public health insurance. A history of asthma was present in 78 (19.7%), diabetes in 12 (3.0%), and immunosuppression in 5 (1.3%). Only 12 participants (3.0%) had received a COVID‐19 vaccine. Children with severe outcomes displayed a higher mean BMI, and a higher proportion had a history of diabetes or immunosuppression compared to those with non‐severe outcomes. A smaller proportion of children with severe outcomes had received a COVID‐19 vaccination than children with non‐severe outcomes (Table 1).

**TABLE 1:** Participant characteristics.

Variables	All (*n* = 395)	Severe (*n* = 105)	Non‐severe (*n* = 290)	*p* value*
Age (year, mean [SD])	7.4 (5.9)	8.4 (6.2)	7 (5.8)	0.032
Missing (*n*, [%])	2 (0.5)	0 (0)	2 (0.7)	
Sex (*n*, [%])				0.111
Female	203 (51.4)	47 (44.8)	156 (53.8)	
Male	190 (48.1)	58 (55.2)	132 (45.5)	
Missing	2 (0.5)	0 (0)	2 (0.7)	
Race (*n*, [%])				0.664
American Indian or Alaskan Native	1 (0.3)	1 (1)	0 (0)	
Asian	9 (2.3)	2 (1.9)	7 (2.4)	
Black or African American	215 (54.4)	55 (52.4)	160 (55.2)	
White	123 (31.1)	33 (31.4)	90 (31.0)	
Other	18 (4.6)	6 (5.7)	12 (4.2)	
Unknown/Missing	29 (7.3)	8 (7.6)	21 (7.2)	
Ethnicity (*n*, [%])				0.523
Non‐Hispanic	346 (87.6)	90 (85.7)	256 (88.3)	
Hispanic	34 (8.6)	10 (9.5)	24 (8.3)	
Missing	15 (3.8)	5 (4.8)	10 (3.4)	
Insurance (*n*, [%])				0.102
Private	92 (23.3)	24 (22.8)	68 (23.4)	
Public	292 (73.9)	76 (72.4)	216 (74.5)	
None	6 (1.5)	4 (3.8)	2 (0.7)	
Missing	5 (1.3)	1 (1.0)	4 (1.4)	
History of asthma (*n*, [%])				1.0
No	315 (79.8)	84 (80.0)	231 (79.7)	
Yes	78 (19.7)	21 (20.0)	57 (19.6)	
Missing	2 (0.5)	0 (0.0)	2 (0.7)	
History of diabetes (*n*, [%])				0.019
No	381 (96.5)	98 (93.3)	283 (97.6)	
Yes	12 (3.0)	7 (6.7)	5 (1.7)	
Missing	2 (0.5)	0 (0.0)	2 (0.7)	
Immunosuppressed (*n*, [%])				0.019
No	388 (98.2)	101 (96.2)	287 (99.0)	
Yes	5 (1.3)	4 (3.8)	1 (0.3)	
Missing	2 (0.5)	0 (0.0)	2 (0.7)	
Body mass index (BMI, kg/m^2^, mean [SD])	23.7 (10.6)	27.3 (13.4)	21.2 (7.3)	0.015
Missing (*n*, [%])	180 (45.6)	73 (69.5)	107 (36.9)	
Reception of COVID vaccine, (*n*, [%])				0.005
No	114 (28.9)	20 (19.0)	94 (32.4)	
Yes	12 (3.0)	1 (1)	11 (3.8)	
N/A (underage)	265 (67.1)	84 (80)	181 (62.4)	
Missing	4 (1.0)	0 (0.0)	4 (1.4)	

*Note:* *p* values were determined by comparing severe and nonsevere groups using *t* tests (age, BMI), chi‐squared tests (sex, ethnicity, asthma history, diabetes history, immunosuppressed), or analysis of variance (ANOVA) testing (race, insurance, received COVID‐19 vaccine). Two participants with nonsevere outcomes are not included in this table.

^*^ *p*‐values based on chi‐square tests for dichotomous features, analysis of variance (ANOVA) for categorical features, and Student’s *t*‐tests for continuous features.

### 3.2 Clinical Features, Treatments, and Outcomes

Compared to participants with non‐severe outcomes, a greater proportion of children with severe COVID‐19 reported fever, dyspnea, chills, nausea or emesis and had higher respiratory rates (Table 2). Children with severe outcomes displayed lower absolute lymphocyte count, hemoglobin level, and platelet count, but higher C‐reactive protein level and glucose level compared to their peers with non‐severe outcomes.

**TABLE 2:** Clinical characteristics of participants.

Variables	All (*n* = 395)	Severe (*n* = 105)	Non‐severe (*n* = 290)	*p* value
Time of illness at enrollment (day, mean [SD])	4.4 (4.2)	5.7 (3.6)	3.9 (4.3)	< 0.001
Patient‐reported symptoms (*n* [%])				
Cough				0.733
No	182 (46.1)	47 (44.8)	135 (46.6)	
Yes	211 (53.4)	58 (55.2)	153 (52.7)	
Missing	2 (0.5)	0 (0.0)	2 (0.7)	
Fever				0.002
No	182 (46.1)	35 (33.3)	147 (50.7)	
Yes	211 (53.4)	70 (66.7)	141 (48.6)	
Missing	2 (0.5)	0 (0.0)	2 (0.7)	
Dyspnea				< 0.001
No	292 (73.9)	52 (49.5)	240 (82.7)	
Yes	101 (25.6)	53 (50.5)	48 (16.6)	
Missing	2 (0.5)	0 (0.0)	2 (0.7)	
Headache				0.348
No	331 (83.78)	92 (87.6)	239 (82.4)	
Yes	62 (15.7)	13 (12.4)	49 (16.9)	
Missing	2 (0.5)	0 (0.0)	2 (0.7)	
Myalgia				1
No	346 (87.6)	93 (88.6)	253 (87.2)	
Yes	47 (11.9)	12 (11.4)	35 (12.1)	
Missing	2 (0.5)	0 (0.0)	2 (0.7)	
Anosmia				0.455
No	384 (97.2)	104 (99)	280 (96.6)	
Yes	9 (2.3)	1 (1)	8 (2.7)	
Missing	2 (0.5)	0 (0.0)	2 (0.7)	
Chills				0.007
No	373 (94.4)	94 (89.5)	279 (96.2)	
Yes	20 (5.1)	11 (10.5)	9 (3.1)	
Missing	2 (0.5)	0 (0.0)	2 (0.7)	
Fatigue				0.349
No	368 (93.2)	96 (91.4)	272 (93.8)	
Yes	25 (6.3)	9 (8.6)	16 (5.5)	
Missing	2 (0.5)	0 (0.0)	2 (0.7)	
Nausea or emesis				0.002
No	265 (67.1)	58 (55.2)	207 (71.4)	
Yes	128 (32.4)	47 (44.8)	81 (27.9)	
Missing	2 (0.5)	0 (0.0)	2 (0.7)	
Diarrhea				0.174
No	325 (82.3)	82 (78.1)	243 (83.8)	
Yes	68 (17.2)	23 (21.9)	45 (15.5)	
Missing	2 (0.5)	0 (0.0)	2 (0.7)	
Abdominal pain				0.338
No	334 (84.6)	86 (81.9)	248 (85.5)	
Yes	59 (14.9)	19 (18.1)	40 (13.8)	
Missing	2 (0.5)	0 (0.0)	2 (0.7)	
Skin rash				0.172
No	367 (92.9)	95 (90.5)	272 (93.8)	
Yes	26 (6.6)	10 (9.5)	16 (5.5)	
Missing	2 (0.5)	0 (0.0)	2 (0.7)	
Vital signs				
Temperature (celsius, mean [SD])	36.6 (0.6)	36.2 (0.3)	36.7 (0.6)	< 0.001
Missing (*n* [%])	4 (1.0)	0 (0.0)	4 (1.4)	
Heart rate (beats/min, mean [SD])	102.7 (24.8)	99 (25.5)	104.1 (24.5)	0.057
Missing	12 (3.0)	3 (2.9)	9 (3.1)	
Respiratory rate (breaths/min, mean [SD])	26.4 (9.5)	29 (11.5)	25.4 (8.5)	0.002
Missing (*n* [%])	14 (3.5)	2 (1.9)	12 (4.1)	
Systolic blood pressure (mm HG, mean [SD])	107.9 (13.7)	108.3 (13.5)	107.7 (13.9)	0.516
Missing (*n* [%])	94 (23.8)	17 (16.2)	77 (26.6)	
Diastolic blood pressure (mm HG, mean [SD])	64.3 (13.3)	64.8 (11)	64.2 (14.1)	0.918
Missing (*n* [%])	94 (23.8)	17 (16.2)	77 (26.6)	
Pulse oximetry (%, mean [SD])	98.3 (1.9)	97.8 (2.1)	98.7 (1.6)	0.001
Missing (*n* [%])	150 (38.0)	11 (10.5)	139 (47.9)	
Laboratory values				
Day of lab draw (day, mean [SD])	1.1 (2.7)	1.7 (3.6)	0.6 (1.4)	< 0.001
Missing (*n* [%])	197 (49.9)	14 (13.3)	183 (63.1)	
White blood cell count (mean [SD])	7.7 (4.2)	7.5 (4.4)	7.9 (4)	0.315
Missing (*n* [%])	197 (49.9)	14 (13.3)	183 (63.1)	
Absolute lymphocyte count (mean [SD])	2.3 (1.9)	2 (1.9)	2.6 (1.9)	0.001
Missing (*n* [%])	197 (49.9)	14 (13.3)	183 (63.1)	
Absolute neutrophil count (mean [SD])	4.5 (3.2)	4.6 (3.4)	4.3 (3.1)	0.507
Missing (*n* [%])	197 (49.9)	14 (13.3)	183 (63.1)	
Hemoglobin (g/L, mean [SD])	11.9 (2)	11.6 (2.2)	12.1 (1.8)	0.039
Missing (*n* [%])	197 (49.9)	14 (13.3)	183 (63.1)	
Platelet count (mean [SD])	266 (116.4)	246.2 (129.3)	282.9 (101.8)	0.005
Missing (*n* [%])	197 (49.9)	14 (13.3)	183 (63.1)	
C‐reactive protein (mg/L, mean [SD])	23.7 (31)	25.3 (29.2)	22.2 (32.8)	0.021
Missing (*n* [%])	237 (60.0)	26 (24.8)	211 (72.8)	
Creatinine (mg/dL, mean [SD])	0.5 (0.4)	0.6 (0.6)	0.5 (0.2)	0.023
Missing (*n* [%])	185 (46.8)	13 (12.4)	172 (59.3)	
Glucose (mg/dL, mean [SD])	111.7 (61.8)	121.8 (74.4)	103.9 (48.8)	0.014
Missing (*n* [%])	185 (46.8)	13 (12.4)	172 (59.3)	
Disposition				
Admitted to hospital (*n* [%])				< 0.001
No	187 (47.3)	1 (1)	186 (64.1)	
Yes	206 (52.2)	104 (99)	102 (35.2)	
Missing	2 (0.5)	0 (0.0)	2 (0.7)	
Hospital stay (day, mean [SD])	2 (6.4)	5.8 (11.2)	0.5 (1.7)	< 0.001
Missing (*n* [%])	2 (0.5)	0 (0.0)	2 (0.7)	
Treatment (*n* [%])				
Supplemental oxygen (non‐invasive)				< 0.001
None	227 (57.5)	35 (33.3)	192 (66.2)	
Nasal cannula	28 (7.1)	23 (21.9)	5 (1.7)	
Face mask	6 (1.5)	4 (3.8)	2 (0.7)	
BIPAP/CPAP	1 (0.3)	1 (1)	0 (0)	
High flow	39 (9.9)	38 (36.2)	1 (0.4)	
NIPPV	4 (1.0)	4 (3.8)	0 (0)	
Missing	90 (22.7)	0 (0.0)	90 (31.0)	
Mechanical ventilation				< 0.001
No	295 (74.7)	95 (90.5)	200 (69.0)	
Yes	10 (2.5)	10 (9.5)	0 (0)	
Missing	90 (22.8)	0 (0.0)	90 (31.0)	
ECMO				0.118
No	303 (76.7)	103 (98.1)	200 (69.0)	
Yes	2 (0.5)	2 (1.9)	0 (0.0)	
Missing	90 (22.8)	0 (0.0)	90 (31.0)	
Respiratory medication				0.001
No	208 (52.7)	68 (64.8)	140 (48.3)	
Yes	62 (15.7)	36 (34.3)	26 (9.0)	
Missing	125 (31.6)	1 (0.9)	124 (42.7)	
Vasopressor/inotrope therapy				< 0.001
No	254 (64.3)	88 (83.8)	166 (57.2)	
Yes	16 (4.1)	16 (15.2)	0 (0.0)	
Missing	125 (31.6)	1 (1.0)	124 (42.8)	
Steroids				< 0.001
No	172 (43.5)	35 (33.3)	137 (47.2)	
Yes	98 (24.8)	69 (65.7)	29 (10.0)	
Missing	125 (31.7)	1 (1.0)	124 (42.8)	
Remdesivir				< 0.001
No	255 (64.6)	90 (85.7)	165 (56.9)	
Yes	15 (3.8)	14 (13.3)	1 (0.3)	
Missing	125 (31.6)	1 (1.0)	124 (42.8)	
IVIG				< 0.001
No	250 (63.3)	84 (80.0)	166 (57.2)	
Yes	20 (5.1)	20 (19.0)	0 (0.0)	
Missing	125 (31.6)	1 (1.0)	124 (42.8)	

Abbreviations: BIPAP, Bilevel positive airway pressure; CPAP, Continuous positive airway pressure; ECMO, Extracorporeal membrane oxygenation; IVIG, Intravenous immunoglobulin; NIPPV, Non‐invasive positive pressure ventilation.

Over half of participants were admitted to the hospital (*n* = 206, 52.2%), and the average length of hospital stay was 2 ± 6.4 days. A greater proportion of children with severe COVID‐19 were admitted to the hospital and had a longer mean length of hospital stay than those with non‐severe outcomes. Of the 105 participants with severe outcomes, 70 (66.7%) were treated with supplemental oxygen, 69 (65.7%) with steroids, 14 (13.3%) with Remdesivir, and 20 (19.0%) with intravenous immunoglobulin therapy. More than one‐third received respiratory medications such as albuterol (*n* = 36, 34.3%). Few children required mechanical ventilation (*n* = 10, 9.5%) and/or vasopressors (*n* = 16, 15.2%), and only 2 (1.9%) required extra corporeal membrane oxygenation.

### 3.3 microRNAs

**FIGURE 2:**
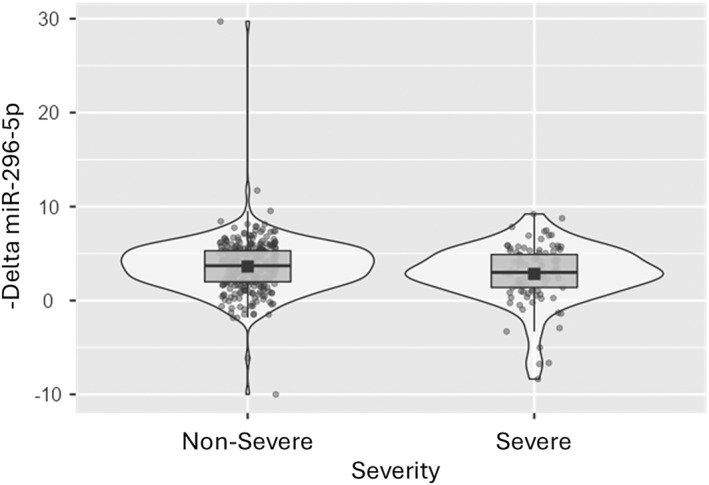
Salivary levels of miR‐296‐5p differ between children with severe and nonsevere SARS‐CoV‐2. The violin plot displays salivary levels of miR‐296‐5p for 395 children with severe (*n* = 105) and nonsevere (*n* = 290) SARS‐CoV‐2 symptoms. Levels of miR‐296‐5p were lower among children with severe SARS‐CoV‐2 symptoms on Wilcoxon testing. In order to promote clinical utility and control for interindividual variation in salivary RNA concentrations, levels of miR‐296‐5p were measured with quantitative polymerase chain reaction and expressed relative to a reference miRNA (miR‐29c‐5p) using the difference in cycles to threshold (−delta Ct) method. Mean levels with standard deviation are shown.

Saliva samples were collected, on average, 4.3 ± 4.2 days after symptom onset. Upon analysis of the saliva samples using qPCR, children with severe outcomes had lower levels of miR‐296‐5p (difference: 0.361, *d* = 0.111, *p* = 0.046) but not miR‐1273c (difference: 0.562, *d* = 0.132, *p* = 0.093) or miR‐4495 (difference: −0.0619, *d* = 0.0145, *p* = 0.826) (Figure 2).

Multivariable adjusted logistic regression analysis demonstrated an inverse association between severe outcomes and mean levels of miR‐296‐5p (adjusted OR = 0.89; 95% CI [0.81, 0.98], *p* = 0.021) while controlling for the previously mentioned covariates (Table 3). A Poisson regression model also demonstrated an association between length of stay in the hospital and mean levels of miR‐296‐5p [adjust RR = 0.91; 95% CI (0.89, 0.92), *p* < 0.001], while controlling for demographic and clinical covariates. A model with stepwise selected list of covariates had an AUC of 0.744 (sensitivity = 0.71, specificity = 0.66, *p* < 0.001) for predicting severe outcomes.

**TABLE 3:** Adjusted of miR‐296‐5p with symptom severity and length of stay.

Variables	OR (95% CI)	*p* value	RR (95% CI)	*p* value
Intercept	0.22 (0.064, 0.78)	0.019	4.44 (3.55, 5.48)	< 0.001
miR‐296‐5p	0.89 (0.81, 0.98)	0.021	0.91 (0.89, 0.92)	< 0.001
Age (0–18 years)	0.41 (0.018, 9.01)	0.574	1.01 (1.00, 1.03)	0.011
Sex (female vs. male)	0.76 (0.45, 1.28)	0.311	1.29 (1.05, 1.38)	< 0.001
Race (AA vs. W)	0.96 (0.51, 1.77)	0.901	1.20 (1.05, 1.38)	0.008
Race (other vs. white)	1.14 (0.49, 2.62)	0.751	0.90 (0.73, 1.10)	0.319
Weight (normal vs. obese)	1.32 (0.70, 2.48)	0.384	0.85 (0.73, 0.98)	0.028
Weight (overweight vs. obese)	0.52 (0.19, 1.45)	0.217	0.50 (0.37, 0.65)	< 0.001
Weight (underweight vs. obese)	0.42 (0.11, 1.58)	0.205	1.23 (0.94, 1.59)	0.116
Asthma (yes vs. no)	1.19 (0.57, 2.52)	0.632	1.76 (1.52, 2.04)	< 0.001
Diabetes (yes vs. no)	3.69 (0.88, 15.50)	0.074	3.94 (3.29, 4.79)	< 0.001
Insurance (public vs. private)	1.23 (0.63, 2.38)	0.535	1.55 (1.31, 1.84)	< 0.001
Insurance (none vs. private)	6.00 (0.88, 40.53)	0.066	1.28 (0.76, 2.04)	0.315
Symptom duration (days)	1.09 (1.03, 1.16)	0.002	1.03 (1.01, 1.04)	< 0.001
Fever (yes vs. no)	1.95 (1.11, 3.41)	0.019	1.57 (1.37, 1.80)	< 0.001

*Note:* The table displays odds ratios (with 95% CI) for a multivariable logistic regression model employing miR‐296‐5p levels and nine known clinical/demographic risk factors for severe SARS‐CoV‐2 symptoms. Relative risk ratios (with 95% CI) for a Poisson multivariable model examining the relationship between miR‐296‐5p levels and length of hospital stay (in days). Both models control for symptom duration (days) at the time of saliva miRNA collection.

## 4 Discussion

This cohort study of children with SARS‐CoV‐2 infection builds upon prior work, demonstrating that saliva miRNA levels may predict severe outcomes in children with SARS‐CoV‐2 infection [45]. Specifically, low levels of miR‐296‐5p were associated with an increased likelihood of severe outcomes and an increased hospital LOS. These relationships remained significant, even when controlling for demographic and clinical covariates.

Although miRNAs have been shown to impact the prognosis of other diseases [20, 21, 22, 23, 24, 25, 26, 27, 28, 29, 30], evidence regarding their role in SARS‐CoV‐2 pathogenesis is limited [32, 33, 34, 35, 36, 37, 38, 39, 40, 41, 42, 43, 44, 45]. In a study comparing serum miRNA levels between 10 adults with SARS‐CoV‐2 infection, 55 miRNAs were found to reflect disease severity [39]. Another study examining blood samples from 96 individuals with SARS‐CoV‐2 identified 200 miRNAs with differential expression [40]. Putative targets of these miRNA biomarkers included immunologic and inflammatory pathways. Our prior study involving RNA sequencing data from 195 children with SARS‐CoV‐2 infection determined that 43 miRNAs were differentially expressed in those with severe outcomes [45]. Specifically, the levels of miR‐296‐5p were downregulated with a twofold difference between those children with and without severe outcomes. This study provides additional evidence that miR‐296‐5p is differentially expressed in the saliva of children with severe outcomes from SARS‐CoV‐2 infection. Saliva is easily collected from young children without the pain and infection risks associated with venipuncture, making it an ideal biofluid for clinical testing [44]. Unlike blood draws, saliva collection does not require specific training, making saliva miRNA testing translatable to outpatient clinics, busy acute care settings, or even at‐home tests, where it could aid critical decisions about patient disposition.

Reductions in circulating miRNA levels have been reported in previous studies of adults with COVID‐19 [39, 40]. These prior studies suggest that the SARS‐CoV‐2 virus may downregulate host miRNAs to escape the host immune system and enhance replication, similar to other viruses [51, 52, 53, 54, 55, 56]. The physiologic relevance of miRNAs in children with severe COVID‐19 is also supported by their role as regulators of immune function and inflammation. Specifically, miR‐296‐5p may play an important role in the interferon cascade after a serious viral infection [53]. In vitro studies of human cells have demonstrated that upregulation of miR‐296‐5p prevents enterovirus 71 (EV71) replication [54] and suppresses inflammatory cascades associated with influenza A virus infection in lung epithelium [55]. A cohort study of individuals with human immunodeficiency virus (HIV)‐1 found that miR‐296‐5p levels were lower in serum compared to persons without HIV‐1 [56]. Together, these studies provide pathophysiologic support for the role of miR‐296‐5p in the host immune response to viral infections.

The prospective design and large numbers of children with severe illness represent strengths of this study. The study's generalizability may be limited in clinical settings that have a lower incidence of severe illness (i.e., nonpandemic conditions). Convenience sampling may have also introduced sampling bias. The central goal of this study was to differentiate severe and nonsevere SARS‐CoV‐2 infection in pediatric patients at the time of ED presentation. For this reason, controls without SARS‐CoV‐2 infection were not included. Enrollment took place from March 2021 to February 2022. During this period, the most common SARS‐CoV‐2 variants were *Delta* and *Omicron*. Generalizability to later variants may be limited. To enhance applicability to clinical settings, saliva was collected at the time of initial clinical presentation to the ED. We controlled for days after symptom onset in our regression models; however, it cannot be assumed that measurement of miRNAs on the day of disease onset would accurately predict illness severity. Similarly, the lack of longitudinal miRNA measurements in this study does not allow us to determine whether repeated miRNA sampling would reflect symptom trajectory. Future studies should assess these timing factors and investigate the relationship between salivary miRNAs and illness severity for other common respiratory illnesses.

## 5 Conclusions

Salivary levels of miR‐296‐5p were lower in children with severe SARS‐CoV‐2 outcomes than in children with nonsevere SARS‐CoV‐2 symptoms. Measuring saliva miRNA in conjunction with clinical characteristics may aid in the prediction of clinical outcomes. Additional longitudinal studies to assess the utility of saliva miRNAs in SARS‐CoV‐2 and other viral infections are needed.

## Author Contributions

**Steven D. Hicks:** methodology, formal analysis, writing–original draft, funding acquisition. **Dongxiao Zhu:** conceptualization, methodology, writing–review and editing, funding acquisition. **Nirupama Kannikeswaran:** investigation, writing–review and editing. **Kathleen Meert:** investigation, writing–review and editing. **Wei Chen:** methodology, formal analysis, data curation, writing–review and editing. **Rhea Sullivan:** investigation, writing–review and editing. **Srinivasan Suresh:** investigation, data curation, writing–review and editing. **Usha Sethuraman:** conceptualization, methodology, investigation, data curation, writing–original draft, project administration, funding acquisition.

## Funding

This research was funded by Eunice Kennedy Shriver National Institute of Child Health and Human Development of the National Institutes of Health through the RADx program under Grant No. R61HD105610 and R33HD105610. The content is solely the responsibility of the authors and does not necessarily represent the official views of the National Institutes of Health.

## Ethics Statement

The study was conducted in accordance with the Declaration of Helsinki and approved by the Institutional Review Board of the University of Pittsburgh (MOD21010046‐003, approval date: 2/25/2021). Informed consent was obtained from all participants.

## Conflicts of Interest

S.D.H. has previously served as a scientific advisory board member for Spectrum Solutions and chief medical officer for Quadrant Biosciences, neither of which played a role in this research. The other authors declare no conflicts of interest.

## Data Availability

Data that support the findings in this study are available from the corresponding author upon reasonable request.
